# 2DProts: database of family-wide protein secondary structure diagrams

**DOI:** 10.1093/bioinformatics/btab505

**Published:** 2021-07-09

**Authors:** Ivana Hutařová Vařeková, Jan Hutař, Adam Midlik, Vladimír Horský, Eva Hladká, Radka Svobodová, Karel Berka

**Affiliations:** National Centre for Biomolecular Research, Faculty of Science, Masaryk University, Brno 625 00, Czech Republic; Centre for Structural Biology, CEITEC—Central European Institute of Technology, Masaryk University, Brno 625 00, Czech Republic; Department of Computer Systems and Communications, Faculty of Informatics, Masaryk University, Brno 602 00, Czech Republic; National Centre for Biomolecular Research, Faculty of Science, Masaryk University, Brno 625 00, Czech Republic; Centre for Structural Biology, CEITEC—Central European Institute of Technology, Masaryk University, Brno 625 00, Czech Republic; National Centre for Biomolecular Research, Faculty of Science, Masaryk University, Brno 625 00, Czech Republic; Centre for Structural Biology, CEITEC—Central European Institute of Technology, Masaryk University, Brno 625 00, Czech Republic; National Centre for Biomolecular Research, Faculty of Science, Masaryk University, Brno 625 00, Czech Republic; Centre for Structural Biology, CEITEC—Central European Institute of Technology, Masaryk University, Brno 625 00, Czech Republic; Department of Computer Systems and Communications, Faculty of Informatics, Masaryk University, Brno 602 00, Czech Republic; National Centre for Biomolecular Research, Faculty of Science, Masaryk University, Brno 625 00, Czech Republic; Centre for Structural Biology, CEITEC—Central European Institute of Technology, Masaryk University, Brno 625 00, Czech Republic; Department of Physical Chemistry, Faculty of Science, Palacký University Olomouc, Olomouc 771 46, Czech Republic

## Abstract

**Summary:**

Secondary structures provide a deep insight into the protein architecture. They can serve for comparison between individual protein family members. The most straightforward way how to deal with protein secondary structure is its visualization using 2D diagrams. Several software tools for the generation of 2D diagrams were developed. Unfortunately, they create 2D diagrams based on only a single protein. Therefore, 2D diagrams of two proteins from one family markedly differ. For this reason, we developed the 2DProts database, which contains secondary structure 2D diagrams for all domains from the CATH and all proteins from PDB databases. These 2D diagrams are generated based on a whole protein family, and they also consider information about the 3D arrangement of secondary structure elements. Moreover, 2DProts database contains multiple 2D diagrams, which provide an overview of a whole protein family's secondary structures. 2DProts is updated weekly and is integrated into CATH.

**Availability and Implementation:**

Freely accessible at https://2dprots.ncbr.muni.cz. The web interface was implemented in JavaScript. The database was implemented in Python.

**Supplementary information:**

Supplementary data are available at *Bioinformatics* online.

## 1 Introduction

Nowadays, there are more than 170 000 structures in Protein Data Bank (PDB) (Armstrong *et al.*, 2020). These structural data enabled the establishment of rich datasets describing individual protein families. Specifically, close to 7000 protein families are listed in the CATH database (Sillitoe *et al.*, 2021), and, for some of them, hundreds to thousands of their member proteins' structures have been determined. These structures originate from different organisms, bind various ligands and contain diverse mutations. These data provide us with a robust basis for examining individual protein families, discovering their essential parts and understanding their structure-function relationships.

A key insight into the structure of a protein is often provided by the visualization of its secondary structures, i.e., the spatial organization of its secondary structure elements (SSEs) such as α-helices and ß-sheets. The most straightforward way to compare protein structures within a protein family might be its visualization using secondary structure 2D diagrams.

Several tools for the 2D visualization of protein secondary structure have been developed (e.g. PROMOTIF; Hutchinson and Thornton, 1996, Pro-origami; Stivala *et al.*, 2011, HERA; Hutchinson and Thornton, 1990). Unfortunately, these tools typically operate with one protein structure. Therefore, two similar proteins from one protein family can have very different 2D diagrams, because they do not consider the global positions of secondary structure elements in space, but only local structure usually within a rectangular grid. These tools are also not able to provide a 2D diagram of multiple secondary structures, e.g., secondary structures of all members of a protein family.

To fill this gap, we have developed the 2DProts database: A comprehensive and up-to-date resource providing secondary structure 2D diagrams for all protein domains from PDB database and multiple 2D diagrams for all protein families from the CATH database.

Main goals of 2DProts are minimization of the error of secondary structure projection from 3D to 2D; highlighting similarities of protein families in each 2D diagram; preservation of differences between protein 3D models within a protein family; and visualization of these differences in 2D diagrams of secondary structures of proteins from such family.

## 2 Algorithm

The algorithm of 2DProts works as follows:

**Input:** A CATH superfamily (e.g. 2.60.120.400), the list of its domains (e.g. 1gztA00, 1ourA00, …), and the PDB structures of these domains.

**Step 1:** For each domain in the given family, find its SSEs (via SecStrAnnotator (Midlik *et al.*, 2019, 2021)) and annotate them in such a way that topologically equivalent SSEs have the same name (via SecStrAnnotator).

**Step 2:** For each group of SSEs with the same name, compute average length and frequency of SSE occurrence.

**Step 3:** For each domain in the family:

*Step 3.1:* Try to select an appropriate starting layout among the previously computed domains.

*Step 3.2:* Group all β-strands into sheets and compute a 2D model of each individual sheet.

*Step 3.3:* Divide the helices and sheets into primary (common for most of the domains) and secondary (the remaining ones).

*Step 3.4:* Place all primary helices and sheets into the 2D diagram.

*Step 3.5:* Adjust the angles of the primary helices and sheets.

*Step 3.6:* Add all secondary helices and sheets into the 2D diagram.

*Step 3.7:* Adjust the angles of the secondary helices and sheets.

**Step 4:** Draw an individual 2D diagram for each domain and a common multiple 2D diagram for the whole family.

Please note, that steps 3.2 to 3.7 employ an optimization algorithm to minimize the error of projection from 3D to 2D and restrict the deviation from the starting layout. Details of the algorithm are described in the Supplementary Material.

## 3 Database contents and functionality

**Fig. 1.**
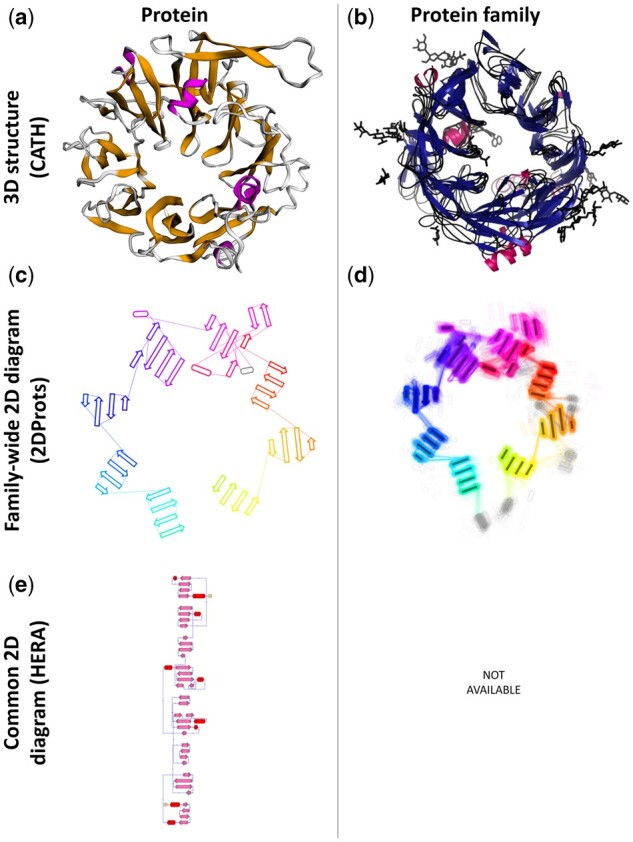
Example of 3D structures and 2D diagrams of a cytochrome reductase domain (PDB ID 1orw, CATH identifier 1orwB01) from protein family with CATH ID 2.140.10.30. Panels show from top left—(**a**) representative 3D structure of the domain (visualization obtained from CATH), (**b**) 3D structures of the all the domains from the family (visualization obtained from CATH), (**c**) representative 2D diagram of the domain from 2DProts, (**d**) multiple 2D diagram of all domains from the family from 2DProts, with SSE averages through protein family and transparency, (**e**) 2D diagram of the domain (visualization obtained from HERA; Hutchinson and Thornton, 1990).

For each PDB structure, 2DProts provides 2D diagrams of all its domains (an example of such a diagram for a protein in Fig. 1a is in Fig. 1c). On top of that, for each protein family, 2DProts provides multiple 2D diagrams of all domains occurring in the family (see example in Fig. 1b and d). In total, 2DProts contains a visualization of more than 400 000 protein domains from all PDB protein structures. Individual protein domains are grouped into about 7000 protein families according to the CATH database. Moreover, 2DProts is able to generate 2D and multiple 2D diagrams also for user-defined protein families. 2DProts database is freely accessible at https://2dprots.ncbr.muni.cz.

The website of the database includes documentation that explains the methodology, user manual for the database, and four examples of scientifically interesting families (porin, cytochrome reductase, cytochrome P450 and methionine sulfoxide reductase). The last example also demonstrates the possibilities of a more detailed clustering.

Each protein domain is represented in the 2DProts database via its identifier in the CATH format (e.g. 1r9nA01), and its 2D diagram is findable using this identifier. It is also possible to search for all domains of a specific protein using its PDB ID (e.g. 1r9n). Protein families can be found via a CATH identifier (e.g. 2.140.10.20) that can be used in the search field to obtain a multiple 2D diagram of the family.

## 4 Discussion

In comparison with other tools generating 2D diagrams of protein secondary structure, 2DProts has the following three marked advantages: Firstly, 2DProts reflects 3D arrangement of SSEs (see Fig. 1c), whereas other tools do not (e.g. see Fig. 1e). Consequently, if some SSEs are close in 3D, they are also close in 2D and vice-versa. Therefore, the 2D visualization is more intuitive. Secondly, 2D diagrams for individual protein family members are intercomparable and can show differences among family members. Thirdly, 2DProts provides multiple 2D diagrams, which can serve as an overview of a domain arrangement within a whole protein family.

Applicability of 2DProts is demonstrated by the fact that 2D diagrams and multiple 2D diagrams from 2DProts were recently integrated into the CATH database (Sillitoe *et al.*, 2021). Its integration into other resources (e.g. PDBe-KB (PDBe-KB consortium, 2020)) is planned. Source code of the website is available at https://gitlab.com/jhutar/2dprot-web.

## Funding

This work was supported by Ministry of Education, Youth and Sports of the Czech Republic under the ELIXIR CZ research infrastructure project, including access to computing and storage facilities [grant number LM2018131]; and European Regional Development Fund—project ELIXIR-CZ [grant number CZ.02.1.01/0.0/0.0/16_013/0001777].

*Conflict of Interest*: none declared.

## Data availability statement

The input data for the 2DProts database are sourced from the P DB database (https://www.ebi.ac.uk/pdbe) and the CATH database (https://www.cathdb.info). The SecStrAnnotator used by 2DProts is available at https://sestra.ncbr.muni.cz. Database 2DProts with all computed 2D diagrams is freely accessible at https://2dprots.ncbr.muni.cz. Source code of the website of 2DProts is available at https://gitlab.com/jhutar/2dprot-web.

## Supplementary Material

btab505_Supplementary_Data
