# Supplementary material for: 2DProts: database of family-wide protein secondary structure diagrams
Source: Bioinformatics. 2021 Jul 9;37(23):4599–601. doi: 10.1093/bioinformatics/btab505 (PMC8652034; doi:10.1093/bioinformatics/btab505)
Supplement: btab505_Supplementary_Data [file btab505_supplementary_data.pdf]

Supplementary Information for 2DProts: Database of Family-Wide Protein Secondary Structure Diagrams

Ivana Hutařová Vařeková[1,2,3], Jan Hutař[1,2], Adam Midlik[1,2], Vladimír Horský[1,2], Eva Hladká[3], Radka Svobodová[1,2,*], Karel Berka[4,*]

[1]National Centre for Biomolecular Research, Faculty of Science, Masaryk University, Brno 625 00, Czech Republic,
[2]CEITEC - Central European Institute of Technology, Masaryk University, Brno 625 00, Czech Republic,
[3]Faculty of Informatics, Masaryk University, Brno 602 00, Czech Republic, and
[4]Department of Physical Chemistry, Faculty of Science, Palacký University, Olomouc 771 46, Czech Republic

Table of Contents

1. Terminology 1
2. Methods 2
Step 1: Detection and annotation of SSEs 2
Step 2: Pre-processing of the CATH superfamily 2
Step 3: Processing of individual domains 2
Step 3.1: Search for cluster start domain 2
Step 3.2: Joining strands into sheets and generating 2D model for each sheet 2
Step 3.3: Division of helices and sheets into primary and secondary 4
Step 3.4: Placing of all primary helices and sheets into the 2D diagram 5
Step 3.5: Adjustment of angles of primary helices and sheets in the 2D diagram 5
Step 3.6: Adding all secondary helices and sheets into the 2D diagram 5
Step 3.7: Adjustment of secondary SSEs angles 6
Step 4: Drawing 2D diagrams 6
3. Bibliography 8

1. Terminology

The 2D diagram is represented as a plane, which contains an x-axis and y-axis. These axes cross in the origin, at the point (0,0).

The following information defines each secondary structure element (SSE) in a 2D diagram:

- the type of SSE (a helix or a strand),
- the name of the SSE (e.g., H1, E2),
- the 2D position of the centre of the SSE in the above plane,
- the angle of the SSE with respect to the x-axis,
- the length of the SSE,
- the colour of the SSE,
- the start and end residue of the SSE, and
- the beta-connectivity between strands in each sheet.

Note: 2DProts can provide this information in a JSON file.[1]

[1] e.g., https://2dprots.ncbr.muni.cz/static/web/layouts/generated-1.10.30.10/layout-4nod_G01.json

In the 2D diagrams, we also introduce one visualization tweak: Fixed ratio of 1:0.2 between the distances between SSEs and the sizes of SSEs. This allows us to place the SSEs comfortably in the 2D diagram without too many overlapping SSEs.

2. Methods

The 2DProts workflow can be described using the following steps:

Step 1: Detection and annotation of SSEs

For a given CATH superfamily, we process the PDB structures of all of its domains. Then, using SecStrAnnotator[2] (Midlik *et al.*, 2019, 2021), we (i) detect all SSEs present in the protein domains and (ii) annotate all topologically equivalent SSEs with the same name.

Step 2: Pre-processing of the CATH superfamily

For each group of SSEs with the same name (e.g., group of helices denoted H5), we compute statistical data, i.e., its average length (measured in the number of residues) and probability of occurrence.

In this step, we also find a universal start domain of the family. First, we choose seven random domains. Then, we compute their mutual RMSD. Finally, we choose the domain with the lowest sum of RMSD with the other domains as the universal start domain for the whole family. When we update the family (and not generate a new diagram from scratch), the existing start domain from the previous version is used instead.

Step 3: Processing of individual domains

The third step is composed of 7 substeps necessary to compute the positions of the SSEs in 2D diagram for a domain and multiple 2D diagrams of the whole protein family.

Step 3.1: Search for cluster start domain

For each domain, we try to find its cluster start domain, i.e., a domain in the same CATH S35 cluster[3] for which the 2D diagram has already been computed. If there is no such domain, we use the universal start domain instead. If the universal start domain has not yet been computed, no start domain is considered.

Step 3.2: Joining strands into sheets and generating 2D model for each sheet

Joining strands into sheets

For each domain, we first sort all of its strands into sheets (i.e., groups of strands that are interconnected by hydrogen bonds) based on connection data that have been computed by the SecStrAnnotator tool. Then, we remove all sheets that only contain strands that are shorter than two residues.

Generating a 2D model for each sheet

For each sheet, we perform the following process as shown in Figure S1:

- **Initial 2D position of strands:** We set the initial 2D position of all the strands to position based on the 2D position of the start domain (if it is present). If it is not present, we set the initial position as (0,0).
- **Rough sheet 2D model:** We use a sequence of optimization algorithms (based on a modified alternating variable method (Korel, 1990)) to compute a "rough" sheet 2D model. In each iteration of the optimization algorithm, we minimize the error of sheet projection to the 3D structure. More precisely, we minimize the error between the minimal strand distance in the 3D structure of the domain and their distance in our 2D diagram. Specifically, we minimize this expression:

---

[2] https://sestra.ncbr.muni.cz

[3] S35 cluster groups domains, guaranteed to share at least 35% sequence identity.

$$\sum_{s_1,s_2 \in S_\beta} |d_{3D}(s_1,s_2) - d_{2D}(s_1,s_2)| \left(L(s_1) + L(s_2)\right) \left(1 - \frac{\min\left(d_{3D}(s_1,s_2), d_{2D}(s_1,s_2)\right)}{40}\right)$$

where S_β is the set of all strands in the sheet; $d_{3D}(s_1,s_2)$ is the minimal distance of strand s_1 and strand s_2 in the 3D structure; $d_{2D}(s_1,s_2)$ is the distance of the positions of s_1 and s_2 in our 2D diagram; and $L(s)$ is the length of strand s (i.e., its number of residues).

The parameter adjusted in each iteration of the optimization algorithm is the upper limit for distances between connected strands. The limit is gradually reduced from up to 30 Å to 4 Å. The starting limit depends on the size of the sheet, on the presence of the start domain 2D diagram, and on the actual results.

Big sheets, whose strands form a barrel conformation (e.g., families 2.40.160.10 and 2.160.20.20) and have no start domain 2D diagram available, need to have a high limit value to reach a similar shape to that of the 3D structure. With a stricter limit, the expected shape is either not reached, or is more time-consuming to reach.

Small sheets with 3 to 6 strands need fewer optimization algorithm steps with lower limit values to reach their expected shape.

- **Improved sheet 2D model:** When this procedure is finished, we have a rough 2D diagram of the whole sheet. In the source 3D structure, distances of neighbouring strands in sheets are typically between 2–3 Å. However, after processing, our 2D diagram model distances are between 0–4 Å. To improve the diagram and to make the sheet 2D diagram more realistic, we iterate the optimization algorithm once more.

  In this iteration, we choose one main direction of our sheet 2D model and optimize the coordinates of all strands in a way that sets the distance between each connected neighbour to be around 2.5 Å.

- **Final sheet 2D model:** Afterwards, we set the angle of the sheet. All strands in a sheet are either parallel or anti-parallel. They are all orthogonal to the main direction of the sheet. We therefore have to choose from two possible reflections of the whole strand. If we have a start 2D diagram, we choose the position that bears a stronger similarity to the one in the start domain. Otherwise, we choose the position that better represents the 3D structure of the SSEs.

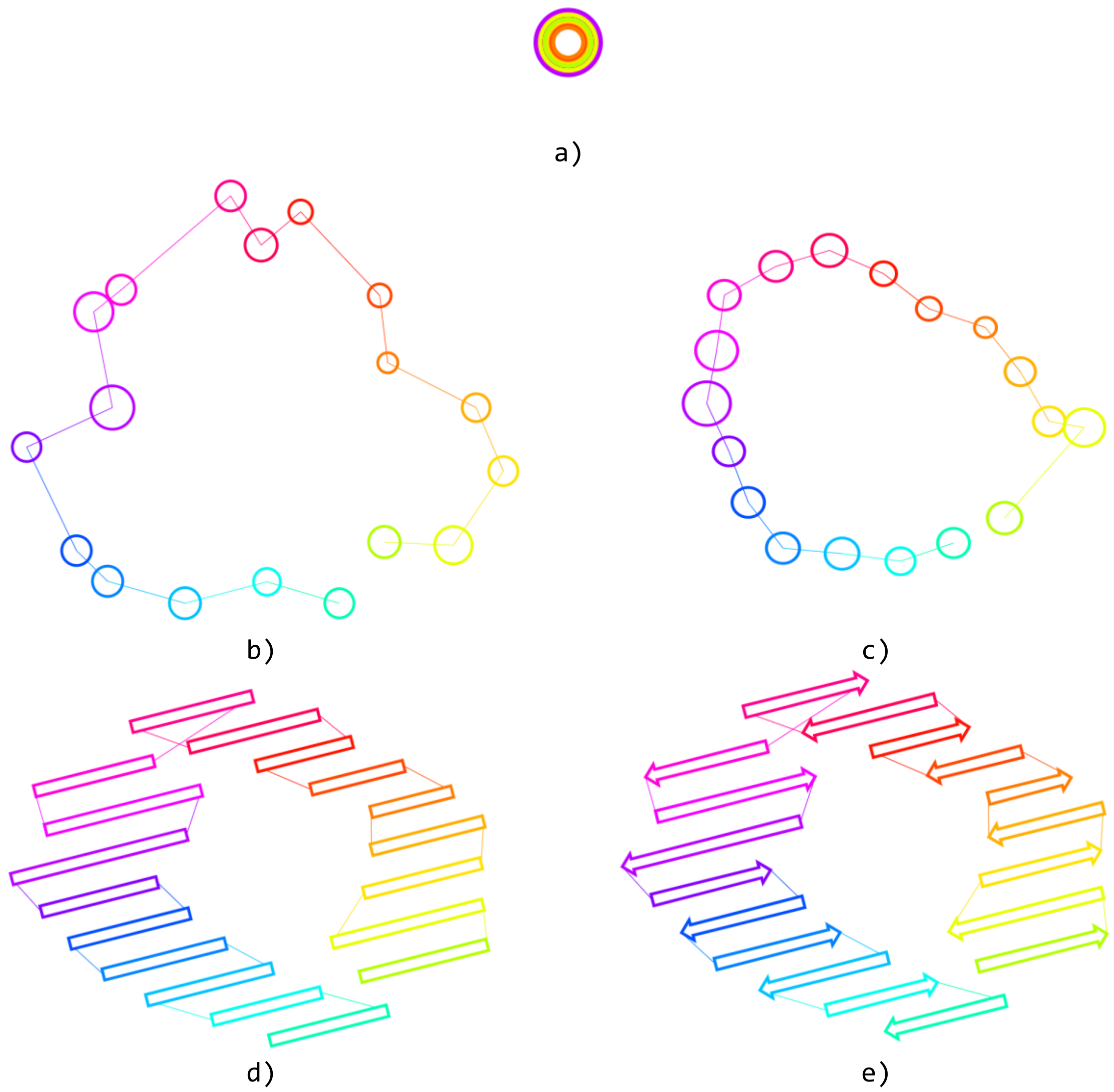

Figure S1: Example of generating sheet 2D model of barrel 3rbhA00 domain (a barrel with 18 strands): a) initial 2D position of strands in origin, b) rough sheet 2D model (first iteration), c) rough sheet 2D model (last iteration), d) improved sheet 2D model, e) final sheet 2D model.

Step 3.3: Division of helices and sheets into primary and secondary

We divide all helices and strands into two sets:

- Primary helices and strands: Common to the majority[4] of domains in the given family
- Secondary helices and strands: Helices and strands which are not primary

The algorithm then divides sheets based on the above strand division:

- Primary sheets contain at least one primary strand
- Secondary sheets do not contain any primary strands.

[4] Majority means they are present in at least 80 % of the domains of the family.

Step 3.4: Placing of all primary helices and sheets into the 2D diagram

As the first step, we place all primary helices and sheets into the 2D diagram. This procedure helps us to make the set of diagrams of the whole family more comparable.

- If there is a start domain, we try to find the positions of primary helices and sheets in the start domain. If there is a helix or sheet position, we use it as the starting position for a given helix or sheet. If not, we set the initial position as (0,0).
- Then, we use an optimization algorithm. In this algorithm, we try to move primary helices and move, rotate, and reflect primary sheets.

During early iterations of the optimization algorithm, we use bigger movement steps and bigger rotation angles. In later iterations, we reduce the length of the movement steps and the rotation angle.

The optimization algorithm minimizes the difference between distances separating the SSEs in the input 3D structure and distances in our 2D diagram. Moreover, to prevent ending at an incomparable minimum[5], we penalize deviation from the start 2D diagram (if there is a start 2D diagram). In this step, we minimize the following function:

$$\sum_{s_1,s_2\in S_\mathrm{p}} |d_{3\mathrm{D}}(s_1,s_2) - d_{2\mathrm{D}}(s_1,s_2)|\,\big(L(s_1)+L(s_2)\big) + \sum_{s\in S_\mathrm{p}\cap S_0} |r(s)-r_0(s)|\,L(s)\frac{|S_\mathrm{p}|}{20}$$

where S_p is the set of all primary SSEs (helices and sheets) of the current domain; S_0 is the set of all SSEs in the layout of the start domain (or empty set if there is no start domain); $d_{3\mathrm{D}}(s_1,s_2)$ is the minimal distance of SSEs s_1 and s_2 in the 3D structure; $d_{2\mathrm{D}}(s_1,s_2)$ is the distance of the positions of s_1 and s_2 in our 2D diagram; $L(s)$ is the length of SSE s (i.e., for a helix the number of its residues, for a sheet the total number of residues in all its strands); $r(s)$ is the 2D position of SSE s in the layout of the current domain; and $r_0(s)$ is the 2D position of SSE s in the layout of the start domain.

Step 3.5: Adjustment of angles of primary helices and sheets in the 2D diagram

As the next step, we optimize the angle of the primary SSEs. We already set fixed angles for all sheets. However, we need the algorithm to determine the angles of the helices.

- If a helix is in the start domain, we use the angle value from the start domain as the initial value. Otherwise, we set the initial angle to 0 °.
- Next, we use an optimization algorithm to minimize the difference between the angles of SSEs in the input 3D structure and our 2D diagram. Specifically, we minimize the following expression:

$$\sum_{s_1,s_2\in S_\mathrm{p}} |\varphi_{3\mathrm{D}}(s_1,s_2) - \varphi_{2\mathrm{D}}(s_1,s_2)|\,\big(L(s_1)+L(s_2)\big)$$

where S_p is the set of all primary SSEs of the current domain; $\varphi_{3\mathrm{D}}(s_1,s_2)$ is the angle between SSEs s_1 and s_2 in the 3D structure; $\varphi_{2\mathrm{D}}(s_1,s_2)$ is the angle between s_1 and s_2 in the 2D diagram; and $L(s)$ is the length of SSE s (i.e., for a helix its number of residues, for a sheet the total number of residues in all of its strands).

Step 3.6: Adding all secondary helices and sheets into the 2D diagram

At this point, all primary SSEs have their positions. It is then necessary to carry out the same process with the secondary structures with respect to all the primary SSEs, whose positions have already been set.

- First, we find the 2D diagram of secondary helices and sheets in the start domain. Then, if such a 2D diagram exists, we use it as a start 2D diagram. If not, we set position (0,0) to every secondary SSE without a reference 2D diagram in the start domain.

---

[5] For one 3D structure, there could be several 2D diagrams with the same deviation from the original 3D structure but with fundamental differences between them.

- Then, we use an optimization algorithm. In this algorithm, we try to move secondary helices and move, rotate, and reflect secondary sheets. First, we use bigger movement steps. Then, during subsequent iterations, we reduce the length of movement steps and the rotation angle. Thus, the optimization algorithm tries to minimize the difference between distances of SSEs in the 3D structure and our 2D diagram with respect to all primary SSEs with firm positions. Moreover, because we want to prevent ending at an incomparable minimum, we penalize the deviation from the start 2D diagram, if it is present. The minimized expression is:

$$\sum_{s_1 \in S_\mathrm{S}, s_2 \in S} |d_{3\mathrm{D}}(s_1, s_2) - d_{2\mathrm{D}}(s_1, s_2)| \left(L(s_1) + L(s_2)\right) + \sum_{s \in S_\mathrm{S} \cap S_0} |r(s) - r_0(s)|\, L(s) \frac{|S|}{20}$$

where S is the set of all SSEs of the current domain; S_s is the set of all secondary SSEs of the current domain; $d_{3D}(s_1,s_2)$ is the minimal distance of SSEs s_1 and s_2 in the 3D structure; $d_{2D}(s_1,s_2)$ is the distance of the positions of s_1 and s_2 in our 2D diagram; $L(s)$ is the length of SSE s (i.e., for a helix its number of residues, for a sheet the total number of residues in all of its strands); $r(s)$ is the 2D position of SSE s in the layout of the current domain; and $r_0(s)$ is the 2D position of SSE s in the layout of the start domain.

Step 3.7: Adjustment of secondary SSEs angles

This adjustment is done in the following way:

- If a secondary helix is in the start domain, we use the angle of the secondary helix from the start domain as the initial value. Otherwise, the initial value is set to 0 °.
- Then, we use an optimization algorithm to minimize the difference between angles of SSEs in the input 3D structure and our 2D diagram. The minimized expression is:

$$\sum_{s_1 \in S_\mathrm{S}, s_2 \in S} |\varphi_{3\mathrm{D}}(s_1, s_2) - \varphi_{2\mathrm{D}}(s_1, s_2)| \left(L(s_1) + L(s_2)\right)$$

where S is the set of all SSEs of the current domain; S_s is the set of all secondary SSEs of the current domain; $\varphi_{3D}(s_1,s_2)$ is the angle between s_1 and s_2 in the 3D structure; $\varphi_{2D}(s_1,s_2)$ is the angle between s_1 and s_2 in the 2D diagram; and $L(s)$ is the length of SSE s (i.e., for a helix its number of residues, for a sheet the total number of residues in all its strands).

Step 4: Drawing 2D diagrams

The last part of the process is to draw an output image that depicts a 2D diagram of helices and sheets and their angles.

2DProts can draw a 2D diagram for one domain or a multiple 2D diagram of a set of domains, which is either the whole protein family, a CATH S35 cluster, or some other subset.

An example of a 2D diagram and the corresponding 3D structure can be found in Figure S2. Examples of four multiple 2D diagrams together with the input 3D structures are in Figures S3–S6, demonstrating protein families with a β-barrel, a β-propeller, a helix bundle, and an α/β domain, respectively.

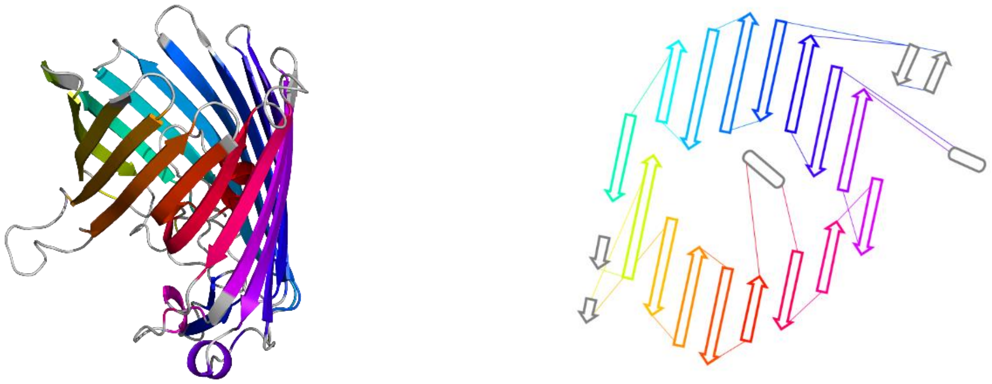

a) b)

Figure S2: The domain 1mpfA00: a) 3D structure, b) 2D diagram from 2DProts.

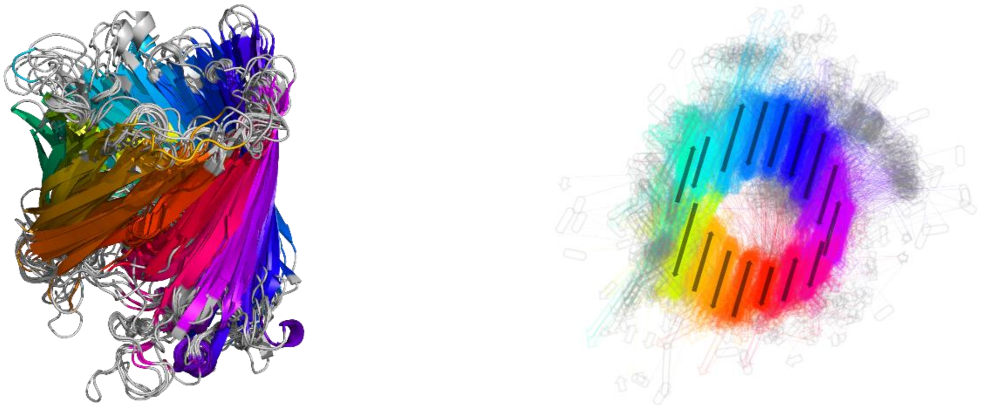

a) b)

Figure S3: Domain family 2.40.160.10, containing a β-barrel. a) 3D structure, b) multiple 2D diagram from 2DProts.

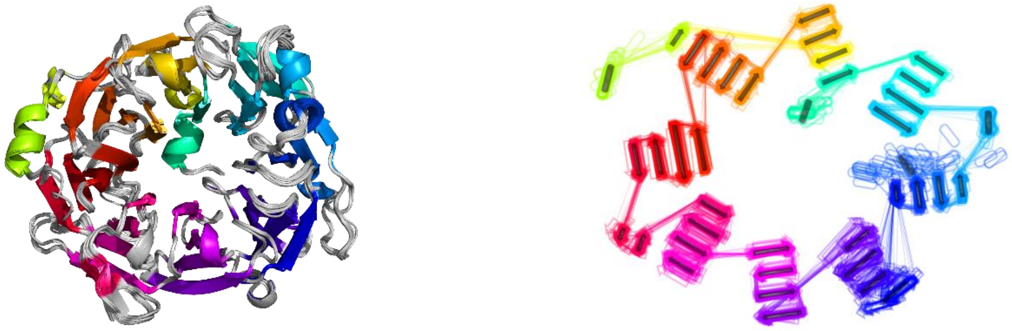

a) b)

Figure S4: Domain family 2.140.10.20, containing 8 β-sheets arranged in a β-propeller. a) 3D structure model, b) multiple 2D diagram from 2DProts.

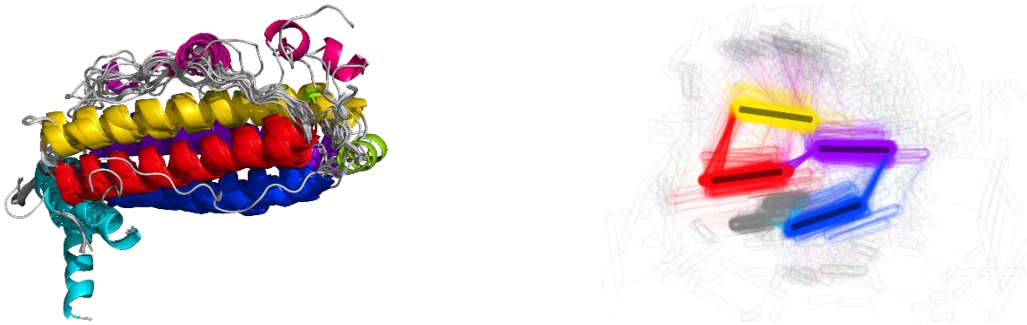

a) b)

Figure S5: Domain family 1.20.1260.10, containing a helix bundle. a) 3D structure, b) multiple 2D diagram from 2DProts.

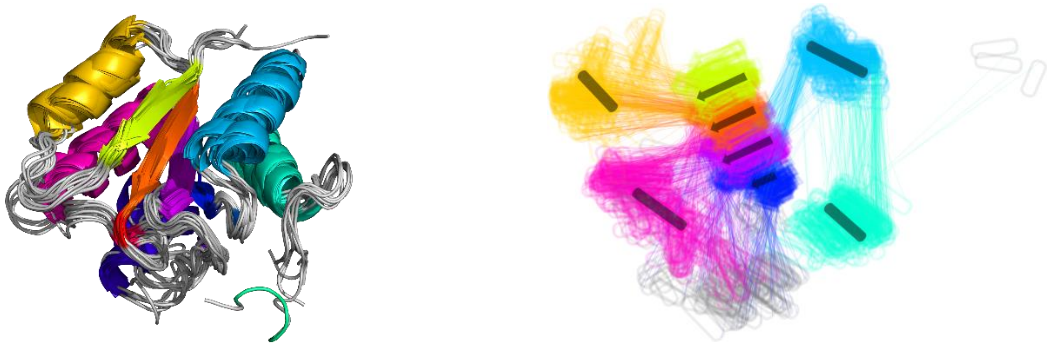

a) b)

Figure S6: Domain family 3.40.50.40, containing an alpha/beta domain. a) 3D structure, b) multiple 2D diagram from 2DProts.

3. Bibliography

Korel,B. (1990) Automated Software Test Data Generation. *IEEE Trans. Softw. Eng.*, **16**, 870–879.

Midlik,A. *et al.* (2019) Automated family-wide annotation of secondary structure elements. In, Kister,A.E. (ed), *Protein supersecondary structures*. Humana Press, New York, pp. 47–71.

Midlik,A. *et al.* (2021) Uncovering of cytochrome P450 anatomy by SecStrAnnotator. *Sci. Rep.*, **11**.
